# Exploring the electromagnetic information of metasurfaces

**DOI:** 10.1093/nsr/nwaa237

**Published:** 2020-09-18

**Authors:** Din-Ping Tsai

**Affiliations:** Department of Electronic and Information Engineering, The Hong Kong Polytechnic University, China

Metasurfaces, a 2D counterpart of metamaterials, are made of planar subwavelength-scale meta-atoms with designed distributions. The meta-atoms of a metasurface can be used to couple incident waves to free space with controllable amplitudes, phases and polarizations, yielding many novel photonic devices such as optical meta-lenses [1–4]. In recent years, with the bloom of information technologies, efforts have been made to braid metasurfaces with digital and information science, rendering the emergence of a digital-coding metasurface, field-programmable metasurface, information metasurface and intelligent metasurface [5–7].

In 2020, Prof. Tie Jun Cui and team members Haotian Wu, Guo Dong Bai, Shuo Liu, Xiang Wan and Qiang Cheng from Southeast University and Prof. Lianlin Li from Peking University brought new physical insights into metasurfaces from an information perspective [8]. In this work, the researchers built on the concept of observation information from the information optics [9] and developed a generalized theory to characterize the information of the digital-coding pattern (${I_1}$) and the far-field pattern (${I_2}$) of metasurfaces. Here, the far-field information (${I_2}$) of a metasurface is defined as the entropy difference between the normalized radiation function and the uniformly distributed pattern. Subsequently, by leveraging the generalized uncertainty relation between two non-commuting observables [10], it is revealed that the upper bound of the far-field information is determined by the size of the meta-surface and the working frequency (Fig. 1).

**Figure 1.**
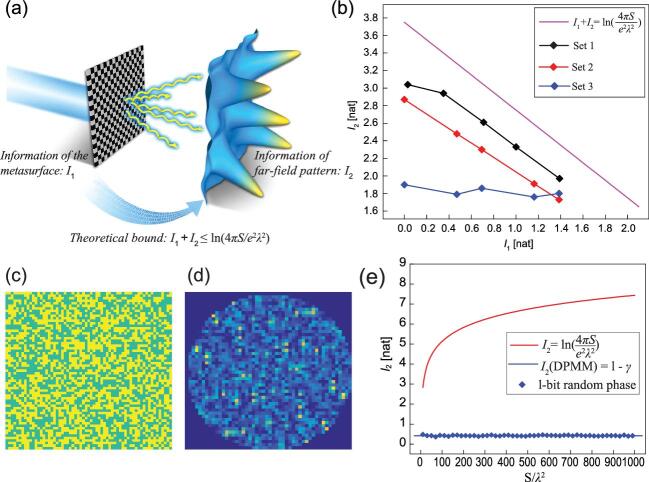
(a) Schematic of the information relationship between the digital-coding metasurface and its far-field pattern. (b) Calculated results of the information relationship between the metasurface samples and their far-field patterns with respect to different modulation schemes and the information upper bound. (c) and (d) A sample of a 1-bit disordered-phase pattern of the metasurface and the generated far-field intensity distribution. (e) Theoretical and calculated results of the far-field information (${I_2}$) with respect to different sizes and phase patterns of metasurfaces. Adapted from Ref. [8].

As an important application, the researchers adopted the established far-field information to predict the upper limit of the number of orthogonal radiation states generated by the digital-coding metasurface, thus providing guidance for metasurface-based computational imaging, for which orthogonal radiation patterns are preferred for compressive-sensing imaging. They explored the information relation between the metasurface and the generated radiation pattern to determine the lower bound of metasurface size as well. Specifically, they demonstrated that, once the required radiation patterns are specified, the size of the metasurface must be larger than the value predicted by the proposed theory; otherwise, it would be impossible to realize the required radiation patterns no matter which design strategies are adopted.

More intriguingly, through investigating the information of a disordered-phase modulated metasurface (DPMM), the researchers found the information-invariance property of chaotic far-field patterns. That is to say, the far-field information of a DPMM is always equal to 1 – γ (γ is the Euler's constant, γ ≈ 0.5772), which is independent of the size of the metasurface, the number of meta-atoms and the phase patterns. The obtained far-field information of a DPMM is close to zero and might be the theoretical lower bound, which indicates that DPMMs are preferred for stealth applications.

The proposed electromagnetic-information theory has considered both the digital world (digital-coding pattern) and the physical world (far-field pattern) and hence will have novel applications on new information theory for 5G and 6G wireless communications.

## FUNDING

This work was supported by the Shenzhen Science and Technology Innovation Commission (SGDX2019081 623281169).

***Conflict of interest statement.*** None declared.
